# Assessment of community healthcare providers ability and willingness to respond to emergencies resulting from bioterrorist attacks

**DOI:** 10.4103/0974-2700.55808

**Published:** 2010

**Authors:** Jeffery S Crane, James D McCluskey, Giffe T Johnson, Raymond D Harbison

**Affiliations:** Center for Environmental/Occupational Risk Analysis and Management, College of Public Health, University of South Florida, Tampa, Florida, USA

**Keywords:** Bioterrorism, emergency preparedness planning, emergency response

## Abstract

**Introduction:**

Previous findings have demonstrated that preparedness and planning within the public health system are inadequately developed to respond to an act of biological or chemical terrorism.

**Materials and Methods:**

This investigation used Internet-based surveys to assess the level of preparedness (PL) and willingness to respond (WTR) to a bioterrorism attack, and identify factors that predict PL and WTR among Florida community healthcare providers. Invitations were sent to 22,800 healthcare providers in Florida, which resulted in 2,279 respondents.

**Results:**

Respondents included physicians (n=604), nurses (n=1,152), and pharmacists (n=486). The results indicated that only 32% of Florida healthcare providers were competent and willing to respond to a bioterrorism attack, 82.7% of providers were willing to respond in their local community, and 53.6% within the State. Respondents were more competent in administrative skills than clinical knowledge (62.8% vs. 45%). Areas in which respondents had the highest competency were the initiation of treatment and recognition of their clinical and administrative roles. Areas in which respondents showed the lowest competency were the ability to identify cases and the ability to communicate risk to others. About 55% of the subjects had previous bioterrorism training and 31.5% had conducted emergency drills. Gender, race, previous training and drills, perceived threats of bioterrorism attack, perceived benefits of training and drills, and feeling prepared were all predictors of overall preparedness.

**Conclusions:**

The findings suggest that only one-third of Florida community healthcare providers were prepared for a bioterrorism attack, which is an insufficient response rate to effectively respond to a bioterrorism incident.

## INTRODUCTION

In the State of Florida, current response plans rely on large numbers of independent, licensed healthcare providers to diagnose and treat the exposed population following a biological weapon attack. This reliance upon the private sector is due to the limited number of government-employed healthcare providers. The planning methodology advocated by the Florida Department of Health at the time of this study required an average of >97% of the licensed healthcare providers to come from the local community in order to activate the county emergency management plans' (CEMP) strategic national stockpile and mass casualty attachments. For example, the Dade County Health Department serves 2.25 million residents with 864 employees, of which ∼23% are licensed medical professionals (physicians, nurses, and pharmacists). In a large-scale biological event, Dade County Health Department's plan would require 15,589 persons with 10,048 being core licensed medical personnel to administer smallpox vaccinations to its population. This is a shortfall of 14,725 total personnel and 9,849 in core medical personnel. The State of Florida would require 117,846 total persons and 75,968 core medical personnel.[1–5] The potential health outcomes from a biological attack require specific training to ensure that healthcare providers are adequately skilled to respond to such incidents. In addition, responding to an incident could affect the provider by exposing him/her to the prevailing condition as well as by ensuing social disruption following a biological attack. The purpose of this study is to identify healthcare providers' level of preparedness, to determine factors that predict the community healthcare providers' clinical and administrative competency (AC) to manage a bioterrorism attack, and to predict their willingness to respond to a biological terrorism attack.

### Materials and Methods

Three primary outcome domains were examined: first was the willingness to respond to a bioterrorism attack; second described ACs; and the third assessed clinical competencies (CCs). The first domain examined whether the provider was willing to respond to a high-risk event and/or a low-risk event, and at what distance from the normal workplace. This assessment used a modified interpretation of the theory of reasoned action (TRA) to help model an individual's ‘willingness to respond’. According to TRA, the most important determinant of the behavior is a person's behavioral intention, in this case, willingness to respond.[6,7] The direct determinants of an individual's behavioral intention (willingness) are attitudes toward performing the behavior (responding) and the subjective norm (perceived belief of professionals performing the behavior).[8] In this study, we looked at the behavioral intentions in the issues of perceived threats/benefits for responding, the perceived ability to successfully respond, and the perceived level of risk to the responders with various demographic factors. While TRA has not been directly used to explain the willingness to respond in an emergency (e.g., hurricane or bioterrorism), it has been used in predicting and explaining a wide range of health behaviors including clinical breast examinations, contraceptive use, drinking, mammography use, smoking, seat belt use, and safety helmet use.[9]

The second domain examined AC of the healthcare providers. This framework was developed using Public Health Workers' Emergency Preparedness Core Competencies for Emergency Response and Bioterrorism initially defined by the Columbia University School of Nursing Center for Health Policy.[10] These competency sets were chosen as the base template for the determination of the bioterrorism competency level (BCL) because of its current integration into Florida's public healthcare system and because of its recognition by the Centers of Disease Control (CDC).[10] Additionally, it is apparent that during an actual bioterrorism response, community healthcare providers would need to be integrated within Florida's public healthcare system. The third domain examined the CC levels of the healthcare providers. This domain was developed using the Emergency Response Clinician Competencies in Initial Assessment and Management produced by the Association of Teachers of Preventive Medicine, in collaboration with Columbia University School of Nursing Center for Health Policy, and 17 national associations, including the American Medical Association (AMA).[10]

In addition to the three domains, we examined the individual demographics of community providers, including age, gender, race, highest educational degree, years worked as a licensed professional, current position, employment status, and work duties. We also obtained workplace demographics such as workplace zip code, patient encounter volume, city type, population size, workplace type, and the existence of a disaster plan at the workplace. Perceived benefits and threats were used to examine the providers' beliefs regarding the benefits of preparedness training, whether their community was at risk for a bioterrorism attack, and whether they had the ability to respond to such an event. Finally, the different types of training methods and their ability to affect the overall preparedness level of healthcare providers were examined. The training types used in this study were grouped as (1) traditional lecture format; (2) online interactive; (3) webcasts, teleconferences, or satellite broadcasts; and (4) self-learn, self-paced study. These factors were included in a stepwise regression model to identify predictive factors of providers' preparedness levels among the surveyed healthcare providers.

## RESULTS

### Distribution of the questionnaire

Of 22,800 questionnaire invitations sent to Florida healthcare providers by e-mail, 9,124 were assumed delivered and 13,676 were returned. There were 2,879 healthcare providers who came to the study website. Of those, 2,279 opted for the study (24.97%). The website survey was open for 7 days and a reminder was sent every 2 days in that period. Of those who opted for the study, 84.9% completed the 59-question survey. All question data were captured up to the point the subjects completed or prematurely exited the survey.

### Description of the study subjects

Categorization of survey participants [Table 1] revealed that 1,152 (50.5%) were nurses, 604 (26.5%) were physicians, 486 (21.3%) were pharmacists, and 37 (1.6%) were “others” (e.g., professor). Over half were (n=1,275) female and most were in 35–54 years of age range (60.5%). Only 55 (2.5%) African-Americans and 139 (6.4%) Hispanics participated in the study. The study population contained an adequate representation of all work experience categories.

**Table 1:** Surveyed Florida healthcare provider demographics

	All (%)	Physicians (%)	Nurses (%)	Pharmacy (%)	Others (%)
Age (n=2,198)					
18–34 years	371 (16.9)	63 (10.4)	163 (15.2)	142 (29.2)	3 (8.3)
35–54 years	1,329 (60.5)	345 (50.7)	687 (64.0)	276 (56.8)	22 (61.1)
>55 years	498 (22.6)	196 (32.5)	223 (20.8)	68 (14.0)	11 (30.6)
Gender (n=2,188)					
Male	913 (41.7)	483 (80.1)	158 (14.9)	263 (54.1)	9 (25)
Female	1275 (58.3)	120 (19.9)	905 (85.1)	223 (45.9)	27(75)
Race (n=2,182)					
African American	55 (2.5)	11 (1.8)	21 (2)	22 (4.5)	1 (2.8)
American Indian	8 (0.4)	4 (0.7)	3 (0.3)	1 (0.2)	0 (0)
Asian/Pacific Island	108 (4.7)	39 (6.5)	31 (2.9)	38 (7.8)	0 (0)
Caucasian	1801 (82.5)	462 (76.6)	944 (89.3)	361 (74.3)	34 (94.4)
Hispanic	139 (6.4)	60 (10)	37 (3.5)	41 (8.4)	1 (2.8)
Other	71 (3.3)	27 (4.5)	21 (2)	23 (4.7)	0 (0)
Highest degree (n=2,184)					
Associate	288 (13.2)	0 (0)	281 (26.5)	0 (0)	5 (13.9)
Bachelor	544 (24.9)	0 (0)	302 (28.5)	233 (48.1)	8 (22.2)
Masters	463 (21.2)	0 (0)	416 (39.3)	34 (7.0)	11 (30.6)
Doctorate	852 (39)	594 (98.5)	38 (3.6)	212 (43.6)	12 (33.3)
Foreign Educated	37 (1.7)	9 (1.5)	22 (2.1)	6 (1.2)	0 (0)
Years of work experience (n=2,168)					
< 2	76 (3.5)	34 (5.6)	22 (2.1)	20 (4.1)	0 (0)
3 to 5	206 (9.5)	52 (8.6)	77 (7.4)	75 (15.4)	2 (5.6)
6 to 10	323 (14.9)	77 (12.8)	146 (14)	94 (19.3)	6 (16.7)
11 to 20	542 (25)	156 (25.9)	254 (24.4)	120 (24.7)	12 (33.3)
> 20	1021(47)	284 (47.1)	544 (52.2)	176 (36.2)	16 (44.4)

*N = NUMBER OF RESPONDERS FOR EACH QUESTION

### Description of the subjects' work place

Most subjects worked in a healthcare setting [Table 2]. The only exceptions were pharmacists whose primary work place was in a community pharmacy. There was also a total of 230 retirees who participated in the survey, though it was unknown whether the retirees continued to practice.

**Table 2:** Surveyed Florida healthcare providers' work place demographics

	All (%)	Physicians (%)	Nurses (%)	Pharmacy (%)	Others (%)
Work place setting (n=2,162)					
Healthcare	1863 (86.2)	530 (88)	895 (86.1)	431 (89)	7 (19.4)
Non-healthcare	179 (8.3)	29 (4.8)	91 (8.1)	38 (7.9)	21 (58.3)
Unemployed	120 (5.6)	43 (7.1)	54 (5.2)	15 (3.1)	8 (22.3)
Primarily work place (n=1,862)					
Hospital					
Nonteaching	470 (25.2)	96 (18.1)	295 (32.9)	76 (17.7)	3 (42.9)
Teaching hospital	331 (17.8)	108 (20.4)	177 (19.8)	46 (10.7)	0 (0)
Long-term care	47 (2.5)	1 (.2)	30 (3.3)	16 (3.7)	0 (0)
Home healthcare	37 (2)	1 (.2)	30 (3.3)	6 (1.4)	0 (0)
Private single pract.	145 (7.8)	103 (19.5)	42 (4.7)	0 (0)	0 (0)
Private multiphysician	179 (9.6)	107 (20.2)	69 (7.7)	2 (.5)	1 (14.3)
Clinic setting	158 (8.5)	53 (10)	88 (9.8)	17 (4)	0 (0)
Institutional pharmacy	21 (1.1)	0 (0)	1 (.1)	20 (4.7)	0 (0)
Community pharmacy	197 (10.6)	0 (0)	0 (0)	197 (45.8)	0 (0)
University/research	47 (2.5)	14 (2.6)	28 (3.1)	2 (.5)	3 (42.9)
Retired	230 (12.4)	46 (8.7)	136 (15.2)	48 (11.2)	0 (0)
Yearly patient encounters (n=1,848)					
< 5000	558 (30.2)	200 (38)	289 (32.5)	68 (16)	1 (14.3)
5,000–9,999	291 (15.7)	99 (18.8)	139 (15.7)	51 (12)	2 (28.6)
10,000–19,999	206 (11.1)	53 (10.1)	97 (10.9)	56 (13.1)	0 (0)
20,000–39,999	202 (10.9)	49 (9.3)	97 (10.9)	53 (12.4)	3 (42.9)
40,000–59,999	147 (8)	19 (3.6)	75 (8.4)	53 (12.4)	0 (0)
60,000–79,999	83 (4.5)	17 (3.2)	43 (4.8)	22 (5.2)	1 (14.3)
> 80,000 223	56 (10.6)	91 (10.2)	76 (17.8)	0 (0)	
Not applicable 138	34 (6.5)	57 (6.4)	47 (11)	0 (0)	
Community type (n=2,128)					
Rural	244 (11.5)	56 (9.5)	125 (12.2)	56 (11.8)	7 (19.4)
Urban	1084 (50.9)	310 (52.5)	522 (50.9)	229 (48.2)	23 (63.9
Suburban	800 (37.6)	225 (38.1)	379 (36.9)	190 (40)	6 (16.7)
Population size (n=2124)					
Small city (< 25,000)	213 (10)	44 (7.5)	103 (10.1)	63 (13.3)	3 (8.3)
Med city (25,000–75,000)	615 (29)	140 (23.7)	332 (32.4)	133 (28.1)	10 (27.8)
Large city (> 75,000)	1296 (61)	406 (68.8)	589 (57.5)	278 (58.6)	23 (63.9)

N IS BASED ON THE NUMBER OF COMPLETION FOR EACH QUESTION

### Administrative competencies

Nurses had a higher AC level than the physicians and pharmacists. Generally, healthcare providers felt most competent at demonstrating the correct use of communication equipment used for emergency communication, and being able to describe their functional role(s) in emergency response, and partaking in these role(s) during regular drills [Table 3]. The findings also suggest that most subjects could problem solve creatively and apply flexible thinking to unusual challenges within their functional responsibilities during a response to a bioterrorism event. Physicians and pharmacists were weakest at identifying limits to own knowledge, skill, and authority, and identify key system resources for referring matters that exceed these limits. The weakness of nurses was their lack of knowledge of their work place's role in an emergency response.

**Table 3:** Administrative competency levels of surveyed Florida healthcare providers

	All healthcare providers (%)	Physicians (%)	Nurses (%)	Pharmacy (%)
AC1	47.7	46.8	51.9	40.3
AC2	56.1	47.6	64.6	49.3
AC3	57.1	47.6	68.2	46.3
AC4	70.1	72.3	69.3	70.1
AC5	72.7	76.3	74.3	66.2
AC6	67.7	65.3	70.5	64.1
AC7	46.1	45.2	56.6	24.7
AC8	70.6	71.3	66.5	78.5

AC1: DESCRIBE YOUR WORK PLACE'S ROLE IN AN EMERGENCY RESPONSE, AC2: IDENTIFY THE CHAIN OF COMMAND IN EMERGENCY RESPONSE, AC3: IDENTIFY AND LOCATE THE AGENCY'S EMERGENCY MANAGEMENT PLAN, AC4: DESCRIBE THEIR FUNCTIONAL ROLE(S) IN EMERGENCY RESPONSE AND PARTICIPATE IN THESE ROLE(S) DURING REGULAR DRILLS, AC5: DEMONSTRATE THE CORRECT USE OF COMMUNICATION EQUIPMENT USED FOR EMERGENCY COMMUNICATION (PHONE, FAX, RADIO, SATELLITE PHONE), AC6: ABILITY TO LOCATE THE COMMUNICATION ROLE(S) IN THE EMERGENCY RESPONSE PLAN AND UNDERSTAND THEIR ROLE, AC7: IDENTIFY LIMITS TO OWN KNOWLEDGE, SKILL, AND AUTHORITY, AND IDENTIFY KEY SYSTEM RESOURCES FOR REFERRING MATTERS THAT EXCEED THESE LIMITS, AC8: DEMONSTRATE CREATIVE PROBLEM SOLVING AND FLEXIBLE THINKING TO UNUSUAL CHALLENGES WITHIN THEIR FUNCTIONAL RESPONSIBILITIES TO RESPOND TO A BIOTERRORISM EVENT.

### Clinical competencies

Physicians had a higher competency level than the nurses and pharmacists on the un-weighted CCs [Table 4]. The ‘all provider’ CC levels for the eight individual unweighted competencies range from the low of 17.9% for the ability to initiate patient care within their professional scope of practice and arrange for prompt referral appropriate to the identified condition(s), to the high of 73.9% for the ability to describe their expected clinical role in bioterrorism response for the specific practice setting as a part of the institution or community response. Within the provider subgroups, physicians and pharmacists were the most competent to respond to an emergency within the emergency management system of their practice, institution, and community. Physicians demonstrated deficits in their ability to communicate risks and actions taken, to patients and concerned others clearly and accurately, and in their ability to recognize an illness or injury as potentially resulting from exposure to a biological, chemical, or radiological agent possibly associated with a terrorist event. Nurses reported difficulty in the recognition of unusual events that might indicate an emergency and describe appropriate action. The pharmacist subgroup displayed major deficits in CC3, CC5, CC6, CC7, and CC8 [[Table T0004]].

**Table 4 T0004:** Clinical competency levels of surveyed Florida healthcare providers

	All healthcare providers (%)	Physicians (%)	Nurses (%)	Pharmacy (%)
CC1	73.9	76.5	72.5	73.3
CC2	70.5	76.5	67.2	71.6
CC3	22.6	34.5	18.4	17.3
CC4	61.4	56.4	67.8	54.6
CC5	17.9	25.7	17.4	9.2
CC6	22.8	29.1	22.3	15.9
CC7	46.1	45.2	56.6	24.7
CC8	38.4	47.3	37.3	29.5

CC1: DESCRIBE THEIR EXPECTED CLINICAL ROLE IN BIOTERRORISM RESPONSE FOR THE SPECIFIC PRACTICE SETTING AS A PART OF THE INSTITUTION OR COMMUNITY RESPONSE, CC2: RESPOND TO AN EMERGENCY WITHIN THE EMERGENCY MANAGEMENT SYSTEM OF THEIR PRACTICE, INSTITUTION, AND COMMUNITY, CC3: RECOGNIZE AN ILLNESS OR INJURY AS POTENTIALLY RESULTING FROM EXPOSURE TO A BIOLOGICAL, CHEMICAL, OR RADIOLOGICAL AGENT POSSIBLY ASSOCIATED WITH A TERRORIST EVENT, CC4: ABILITY TO REPORT IDENTIFIED CASES OR EVENTS TO THE PUBLIC HEALTH AUTHORITIES TO FACILITATE SURVEILLANCE AND INVESTIGATION USING THE ESTABLISHED INSTITUTIONAL OR LOCAL COMMUNICATION PROTOCOL, CC5: INITIATE PATIENT CARE WITHIN THEIR PROFESSIONAL SCOPE OF PRACTICE AND ARRANGE FOR PROMPT REFERRAL APPROPRIATE TO THE IDENTIFIED CONDITION(S), CC6: COMMUNICATE RISKS AND ACTIONS TAKEN TO PATIENTS AND CONCERNED OTHERS CLEARLY AND ACCURATELY, CC7: RECOGNIZE AND MANAGE THE PSYCHOLOGICAL IMPACT OF A BIOTERRORISM EVENT ON VICTIMS AND HEALTHCARE PROFESSIONALS, AS APPROPRIATE TO THE EVENT, CC8: RECOGNIZE UNUSUAL EVENTS THAT MIGHT INDICATE AN EMERGENCY AND DESCRIBE, APPROPRIATE ACTION.

### Weighted administrative competency level

To calculate the weighted administrative competency level (ACL) based on the distribution of provider professions and degree of competency within each professional subgroup, the following formula was used:

ACL = (0.103×AC1) + (0.126×AC2) + (0.103×AC3) + (0.159×AC4) + (0.153×AC5) + (0.062×AC6) + (0.103×AC7) + (0.191×AC8) ACL = 0.628

The result was a mean score of 0.628. This suggests that 62.8% of healthcare providers were competent in the administrative core competencies.

### Weighted clinical competency level

To calculate the weighted clinical competency level (CCL) based on the distribution of provider professions and degree of competency within each professional subgroup, the following formula was used:

CCL = (0.113×CC1) + (0.118×CC2) + (0.153×CC3) + (0.110×CC4) + (0.129×CC5) + (0.131×CC6) + (0.106×CC7) + (0.14×CC8) CCL = 0.450

The result was a mean score of 0.450. This suggests that 45.0% of healthcare providers were competent in the clinical core competencies.

### Bioterrorism competency level

To calculate the BCL, the results from both the ACL and CCL above were used in the following formula:

BCL = (0.364×ACL) + (0.636×CCL)

**Figure 1:**
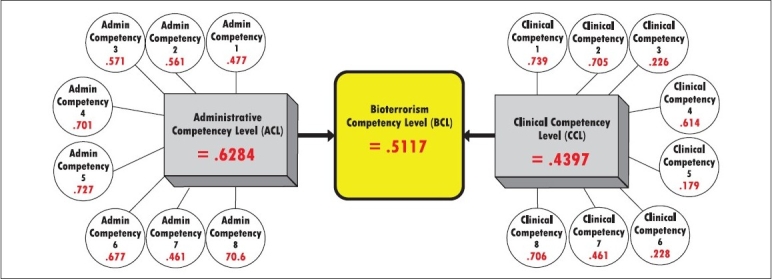
Weighted bioterrorism competency levels scores for surveyed Florida healthcare providers

The result was a mean score of 0.512. This suggests that 51.2% of healthcare providers had the necessary competency level to respond to a bioterrorist attack based on their administrative and CC level. The framework of this determination is shown in Figure 1.

### Willingness-to-respond

The willingness-to-respond score was assessed in terms of the proximity to the incident and the perceived risk of the event. The study results suggest that most Florida providers were willing to respond to both a high-risk (HR) event and a low-risk (LR) event within their local community. Physicians were the most likely to respond to an HR event in the local community, while nurses were the most likely to respond to an LR event. Pharmacists were the least likely to respond in all proximity categories [Table 5].

**Table 5:** Percentage of surveyed Florida healthcare providers willing-to-respond to a bioterrorism attack

	All healthcare providers (%)		Physicians (%)		Nurses (%)		Pharmacy (%)	

Proximity n=1961	High risk	Low risk	High risk	Low risk	High risk	Low risk	High risk	Low risk
Local	81.7	82.8	84.5	83.3	81.6	83.6	79.1	80.7
Regional	64.4	68.1	66.5	65.9	65.5	70.4	59.5	64.6
Statewide	53.6	53.8	55.0	51.7	56.9	56.7	45.0	47.0
Nationwide	48.2	47.0	51.9	46.1	47.3	48.9	45.5	44.5

**Figure 2:**
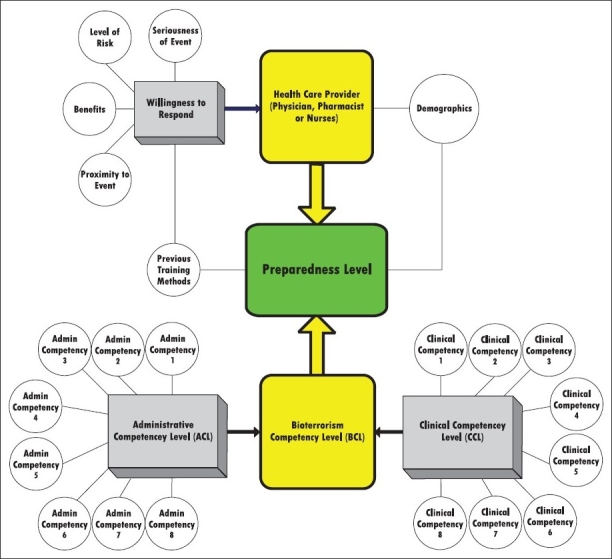
The scored conceptual model for bioterrorism preparedness

When asked if Florida's community healthcare providers were willing to respond to biological agent attacks outside their local community, all subject group percentages dropped dramatically. The derivation of the final weighted preparedness level (0.325) from the overall willingness to respond score and the bioterrorism competency score is illustrated in Figure 2.

The results indicate that approximately 67.5% of Florida healthcare providers could not be adequately utilized in response to a bioterrorism attack. As identified by the BCL, 51.1% of subjects had the minimal competencies needed to respond to a biological attack and 53.7% were willing to respond within the state of Florida [Table 5]. When the process of matching the factors of competency and willingness to respond was applied to the subjects, only 32.5% of Florida's community healthcare providers had both a minimal level of competency to effectively function, and were willing to respond to a bioterrorism attack. Pharmacists seemed to be less prepared than physicians and nurses [Table 6]. This was confirmed with a Pearson chi-square test of the percent preparedness of all three groups, showing that there was a statistically significant difference between the levels of preparedness of the three groups (chi-square =60.916, *P*<0.001). However, there was no statistically significant difference between physicians and nurses, who had similar preparedness scores.

**Table 6:** Preparedness levels of surveyed Florida healthcare providers

Provider type	Overall Preparedness	
	
	Not prepared (%)	Prepared (%)
Physician n=537	351 (65.4)	186 (34.6)
Nurse n=916	564 (61.6)	352 (38.4)
Pharmacist n=436	360 (82.6)	76 (17.4)
FL healthcare providers n=1889	1275 (67.5)	614 (32.5)

The findings suggest that 55.5% (n=1957) of Florida's community healthcare providers did NOT feel prepared and 41.5% felt somewhat prepared to identify and manage a bioterrorism attack. Only 3% of Florida's providers felt very prepared.

### Work place training, planning, and perceptions

The survey indicated that 31.5% of Florida's community healthcare providers had participated in an emergency drill in the last 12 months. Only 11.1% had participated in a bioterrorism themed drill. Survey responses indicate that 55.2% of Florida's community healthcare providers have participated in emergency training sometime during their career and only 11.1% had participated in training within the previous 12 months. As well, 32.3% stated that the training included chemical or biological components.

When asked how important it is for you to be trained to identify a possible bioterrorism attack, 46% of the providers reported very important, 50% stated it was important, and 4% believed it was not important. When asked if a bioterrorism attack is a real threat within Florida, 86.4% of the providers either ‘strongly agreed’ or ‘agreed’. When asked if a bioterrorism attack is a real threat within your local community, this percentage dropped to 59.8% that either ‘strongly agreed’ or ‘agreed’, with 40.2% responded as either being neutral or disagreeing.

### Predictive factors of provider preparedness levels

In the preparedness regression model, if healthcare providers were prepared, the preparedness level became 1 (PL = 1), otherwise PL = 0 (not prepared). The results from logistic regression including all preparedness variables indicated that previous trainings (*P*<0.001) and drills (*P*<0.001) were significant predictors of the overall preparedness level of Florida healthcare providers at 0.05 level.

The results indicate that if a healthcare provider has participated in previous drills, he/she is 2.56 times more likely to be prepared for a bioterrorism attack. Similarly, if a healthcare provider has had previous training, he/she is 2.86 times more likely to be prepared for a bioterrorism attack. Using a similar logistic regression model to evaluate the predictors of ‘willingness to respond’, those who attended previous drills were 1.55 times more likely to be willing to respond to a bioterrorism attack. If the healthcare providers had previous trainings, they were 1.33 times more likely to be willing to respond to a bioterrorism attack. The results from logistic regression show that gender (*P*=0.042), city type (*P*=0.020), current position (*P*=0.033), and primary work place (home healthcare, private single practice setting, or private multiphysician practice; *P*=0.007, 0.005,.000, respectively) were significant predictors of overall preparedness for Florida's healthcare providers. If the healthcare providers were male, they were 1.32 times more likely to be prepared for the bioterrorism attack. If they worked in a rural area, they were 1.52 times more likely to be prepared for the bioterrorism attack than in a suburban area.

## DISCUSSION

### The bioterrorism competency level

The BCL was used to score the overall competency level of the individual and the group as a whole. The BCL uses only the weighted knowledge (competency level) of the providers, not the overall preparedness levels. The BCL in this study suggests that only 51% of Florida's community healthcare providers have the minimum BCL to identify and manage an event without hurting themselves and/or others. The results of this study suggest that providers who have had previous trainings and/or drills were over 2.5 times more likely to be prepared than the providers not trained or drilled.

These results should encourage the FDOH to place emphasis on training community healthcare providers for effective biological agent response. Most bioterrorism training developed prior to this study by FDOH and other entities has been presented at an ‘awareness’ level. This type of training does not better prepare the FDOH emergency planner/coordinator to actually respond to an event. There has been little, if any, operational level training conducted. Unlike hurricanes, the emergency planners/coordinators will not be able to use the standard practice of working through response strategies during the event, without endangering the health and safety of themselves and the population. On the other hand, the administration of operational training to community health providers would greatly increase their preparedness levels. In addition, it could possibly encourage volunteers and be used as a recruitment tool to increase enrollment in the Florida's Medical Reserve Corps (MRC). These providers will be needed to successfully activate the county emergency management plans during a bioterrorism event and/or mass casualty incident.

### The assessment of the providers willingness to respond

This study suggests that most Florida providers were willing to respond to both an HR and LR event within their local community. Alexander and Wynia found that physicians were willing to respond 80% of the time in a lower risk environment, 40% in a higher risk environment, and 33% in the highest risk environment.[11] In this study, 84.5% of physicians reported that they would respond to an HR event and 83.6% would respond to an LR event within their local community. This elevated willingness to respond score in HR events could possibly be explained by the proximity factor.

When a disaster affects friends and family, physicians may be more willing to help, even when the risk is higher. However, when Florida physicians were asked if they were willing to respond to biological agent attacks outside their local community, only 53.6% reported that they are willing to respond to an HR event. While it is still much higher than the previous study, the physicians' willingness level dropped over 20%. The results from this study indicate that previous trainings and drills were significant predictors of the willingness to respond.

### Research strengths

This study was able to collect sufficient data through validated survey methodology to assess the preparedness of a representative sample of healthcare providers in the state of Florida. Statistically significant predictors of preparedness were successfully evaluated through logistic regression to assess the most pertinent needs of the healthcare provider community in promoting response competency.

### Limitations

A larger response rate from healthcare providers would have benefitted this study and given further assurance that the analyzed sample was representative of the healthcare provider community as a whole. Response bias may have been present in the form of an oversampling of those interested in the subject of bioterrorism response, thereby potentially oversampling those who are informed, trained, or otherwise competent in bioterrorism response. As a result of this bias, we may have overestimated the actual preparedness and competency of healthcare providers, which is deficient, as already indicated by our current results.

### Future directions

This study was limited to the State of Florida's healthcare provider community. This analysis should be conducted in every state which employs an emergency response/bioterrorism response plan that relies on the competent participation of nonstate employed healthcare providers. The findings in this study indicate that heavy reliance on private sector healthcare providers may result in an insufficient statewide response to a bioterrorist attack.

## CONCLUSION AND IMPLICATIONS

This study has identified a significant deficit in both Florida healthcare providers' competency and willingness to respond to a bioterrorism attack. This research has also identified predictors of willingness to respond, which includes training and education pertaining to biological agent response and awareness. The implication of these results is that the number of healthcare providers willing to respond to a statewide bioterrorism attack will be insufficient to successfully carry out current response plans developed by the state of Florida. This is compounded by the finding that of the providers willing to respond to such an attack, many of them are not trained to carry out their specific roles in such a response. In addition, the current pandemic influenza response plans, along with the local hospital plans, have been stacked upon the current strategic national stockpile/bioterrorism plans for most counties and states. If a pandemic would require mass immunizations and distribution of medications, and treatment, the same factors assessed within this study would apply.

The results of this study suggest that expanded training of nongovernmental healthcare providers would increase both their competency and willingness to respond to a large-scale bioterrorism incident, which would consequently increase the likelihood of a successful response to a bioterrorism event in the state of Florida. It also suggests that when bioterrorism, Strategic National Stockpile (SNS), and/or a pandemic influenza plans rely on volunteer community healthcare providers to activate and to have a successful emergency response to a bioterrorism incident, the agency may need to review and update its plan. These results may be applicable to other emergency response categories and the survey should be used in these areas to assess preparedness.

## References

[1] Dade County Health Department (2004). Strategic National Stockpile Plan.

[2] EREC (2004). Strategic National Stockpile Plan Template.

[3] Hillsborough County Health Department (2004). Strategic National Stockpile Plan.

[4] Pasco County Health Department (2004). Strategic National Stockpile Plan.

[5] Polk County Health Department (2004). Strategic National Stockpile Plan.

[6] Glanz K, Lewis FM, Rimer BK (1996). Health Behavior and Health Education.

[7] Montano DE, Taplin SH (1996). The Theory of Reasoned Action and the Theory of Planned Behavior. Health Behavior and Health Education.

[8] Ajzen I, Fishbein M (1980). Understanding Attitudes and Predicting Social Behavior.

[9] Fishbein M (1993). The Theory of Reasoned Action: Its Application to AIDS Preventive Behavior.

[10] Gebbie Kristine M (2002). Bioterrorism and Emergency Readiness: Competencies for all Public Health Workers.

[11] Alexander GC, Wynia MK (2003). Ready and willing? Physicians' sense of preparedness for bioterrorism. Health Aff (Millwood).

